# Public Interest in a Potentially Harmful, Non–Evidence-Based “Wellness” Practice: Cross-Sectional Analysis of Perineum Sunning 

**DOI:** 10.2196/24124

**Published:** 2021-01-26

**Authors:** Ryan Ottwell, Micah Hartwell, Tracy Beswick, Taylor Calli Rogers, Heather Ivy, Marcus Goodman, Matt Vassar

**Affiliations:** 1 Oklahoma State University Center for Health Sciences Tulsa, OK United States; 2 Center for Dermatology Tulsa, OK United States; 3 Philadelphia College of Osteopathic Medicine Roswell, GA United States; 4 Goodman Dermatology Roswell, GA United States

**Keywords:** general dermatology, perineum sunning, perineum tanning, skin neoplasm, public health, social media, infodemiology, public interest, Google Trends, Twitter

## Abstract

**Background:**

Perineum sunning/tanning is a potentially harmful yet popular new health trend cultivated by a viral social media post, famous public figures, and subsequent media coverage.

**Objective:**

Our primary objective is to evaluate public interest in perineum sunning/tanning.

**Methods:**

Using an observational study design, we extracted data from Google Trends for the terms “perineum sunning,” “perineum tanning,” “Metaphysical Meagan,” and “Josh Brolin”; and Twitter (via SproutSocial) for “perineum sunning” and “perineum tanning” from November 1, 2019, to December 31, 2019. UberSuggest was used to investigate monthly search volumes and user engagement. We used data from Google Trends and Twitter to construct autoregressive integrated moving average (ARIMA) models to forecast public interest in perineum sunning and perineum tanning had the post on social media never occurred. Next, we performed an integral function to calculate the cumulative increase in “perineum tanning” from the day after the post occurred to the end of the year as the area between the forecasted values and the actual values. Using Welch *t* tests, we compared forecasted and actual values for “perineum sunning” and “perineum tanning” using Twitter and Google Trends data over 1-, 2-, and 4-week periods after the social media post to determine if the increased volumes were statistically significant over time. Lastly, we monitored Google Trends for “perineum sunning” and “perineum tanning” through September 30, 2020, to capture trends during the summer months.

**Results:**

Before the Instagram post went viral, there was no search interest in perineum sunning. ARIMA modeling for perineum tanning forecasted no increase in searches (0.00) if the post had not gone viral, while actual interest conveyed a relative cumulative increase of 919.00% from the day the post went viral through December 31, 2020. The term “perineum sunning” was mentioned on average 804 (SD 766.1) times daily for this 7-day period, which was also significantly higher than predicted (*P*≤.03), totaling 5628 tweets for these 7 days. The increased volume of tweets and relative search interest from Google Trends remained significantly higher for both terms over the 1-, 2-, and 4-week intervals. User engagement showed that nearly 50% of people who searched for “perineum sunning” were likely to click a returned link for more information. Continued observance of search interest in perineum sunning demonstrated interest spikes in the summer months, June and July 2020.

**Conclusions:**

Google Trends and Twitter data demonstrated that one social media post claiming non–evidence-based health benefits of regular sun exposure—without the use of sunscreen—generated significant public interest. Medical journals, dermatologists, and other health care professionals are obligated to educate and correct public misperceptions about viral wellness trends such as perineum sunning.

## Introduction

Social media may positively affect health behaviors or propagate potentially harmful health information [1,2]. On October 21, 2019, posts on the social media platform Instagram boasted that perineum sunning would improve focus, augment hormonal regulation, increase libido, regulate circadian rhythm, and enhance health and longevity. These posts claimed that only 30 seconds of perineum sunning was equivalent to one day’s worth of sun exposure while also recommending against sunscreen use when perineum sunning [3]. The original post went viral in late November via Twitter, and again in December after well-known actor Josh Brolin received media coverage for the severe sunburn to his anogenital area after attempting perineum sunning.

Given the high potential for sunburns and cutaneous cancers resulting from this practice, our primary objective was to investigate the effects of social media and news coverage of perineum sunning on public interest by examining internet search volume, trends, and engagement, using publicly available data. A more informed understanding of the influence of social media on public search interest in potentially harmful practices like perineum sunning may assist dermatologists and medical journals when developing social media strategies to directly combat medical misinformation.

## Methods

Google Trends [4] was used to collect daily relative search interest from November 1, 2019, to December 31, 2019, for “perineum sunning,” “perineum tanning,” “Metaphysical Meagan” (the Instagram user who published the original post), and “Josh Brolin” (who appeared in news stories on December 3, 2019, after getting a severe sunburn while performing this practice). Search interest from Google Trends is provided as a relative measure of total searches from 0-100 estimated from the highest peak within a given time frame. To explore public interest beyond Google Trends, we performed keyword searches for “perineum sunning” and “perineum tanning” occurring on Twitter via SproutSocial [5], a social media analytics platform. We also used UberSuggest [6] to collect monthly internet search volumes and user engagement (defined as a person clicking on the links returned from the search) for the terms “perineum sunning” and “perineum tanning.”

Using Google Trends and Twitter data, we constructed autoregressive integrated moving average (ARIMA) models to forecast predicted values of relative search interest and tweets for the terms “perineum sunning” and “perineum tanning” from November 25, 2019, to the end of the year if the post on social media had not occurred. Next, we calculated the average number of tweets and Google Trends relative search interest for the terms “perineum sunning” and “perineum tanning,” and using Welch's *t* tests, compared them to their respective forecasted values over 1-, 2-, and 4-week periods after the social media post to assess if the increased volumes were statistically significant over time. Using an integral function, we calculated the cumulative area between the forecasted baseline and the actual relative search interest data to provide the relative increased search interest through December 31, 2019, for “perineum sunning” from Google Trends. Lastly, Google Trends was monitored for the terms “perineum sunning” and “perineum tanning” through September 30, 2020, to capture public interest trends during the summer months. All analyses were conducted in R, version 3.2.1 (The R Foundation).

## Results

**Figure 1:**
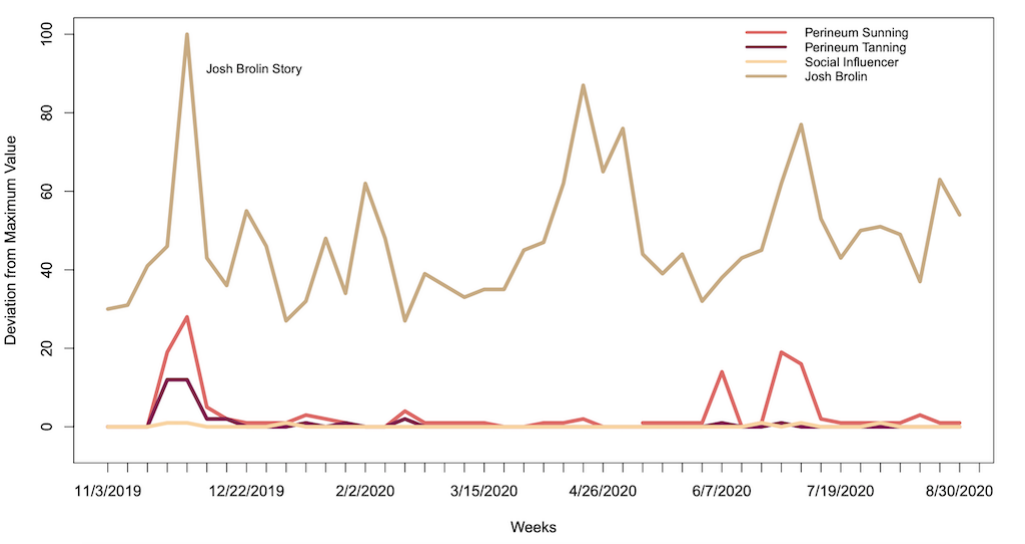
Google Trends analysis of the terms “perineum sunning,” “perineum tanning,” “Metaphysical Meagan,” and “Josh Brolin,” from November 1 to December 31, 2019.

Relative search interest for the four search terms through the end of 2019 are compared in Figure 1.

Based on the first 24 days of November, the ARIMA model forecasted that no interest (0.0) would have arisen in “perineum sunning” for the rest of the year if the social media post had not happened.

Relative search interest for perineum sunning and perineum tanning peaked the day after the social media post went viral. Keyword usage on Twitter showed that tweets significantly increased the day following the post with an increase of 2064 (95% CI 2054-2074) tweets over the predicted value (1) for perineum sunning. For the first 7 days after the post went viral, actual search interest for perineum sunning and perineum tanning were on average 42% (SD 33.0) and 43.6% (SD 35.9) higher than predicted, respectively. Additionally, the term “perineum sunning” was mentioned on average 804 (SD 766.1) times daily for this 7-day period, which was also significantly higher than predicted (*P*≤.03), totaling 5628 tweets for these 7 days. The increased volume of tweets and relative search interest from Google Trends remained significantly higher for both terms over the 1-, 2-, and 4-week intervals (Table 1).

**Table 1:** Differences of means between actual and forecasted data from 1, 2, and 4 weeks after the social media post.

Search term		7 days			14 days			28 days		
		Forecast, mean (SD)	Actual, mean (SD)	*t* test (*df*=6); *P* value	Forecast, mean (SD)	Actual, mean (SD)	*t* test (*df*=13); *P* value	Forecast, mean (SD)	Actual, mean (SD)	*t* test (*df*=27); *P* value
**Google Trends**										
	Perineum sunning^a^ (%)	0 (0)	42.0 (33.0)	–4.2; .001	0 (0)	37.3 (32.4)	–4.3; <.001	0 (0)	21.2 (27.9)	–4.0; <.001
	Perineum tanning^a^ (%)	0 (0)	43.6 (35.9)	–3.2; .02	0 (0)	49.2 (31.3)	–5.9; <.001	0 (0)	30.6 (29.2)	–5.5; <.001
**Twitter**										
	Perineum sunning^b^	2.8 (7.52)	804.1 (766.1)	–2.8; .03	1.4 (5.3)	573.4 (630.5)	–3.4; .004	0.7 (3.8)	299.2 (519.1)	–3.0; .005
	Perineum tanning^b^	0.1 (0.0)	36.1 (37.4)	–2.5; .04	0.1 (0)	27.8 (28.4)	–3.6; .003	0.1(0)	15.0 (23.7)	–3.3; .002

^a^Data reflects average daily relative search interest (0%-100%).

^b^Data reflects average daily number of actual tweets.

**Figure 2:**
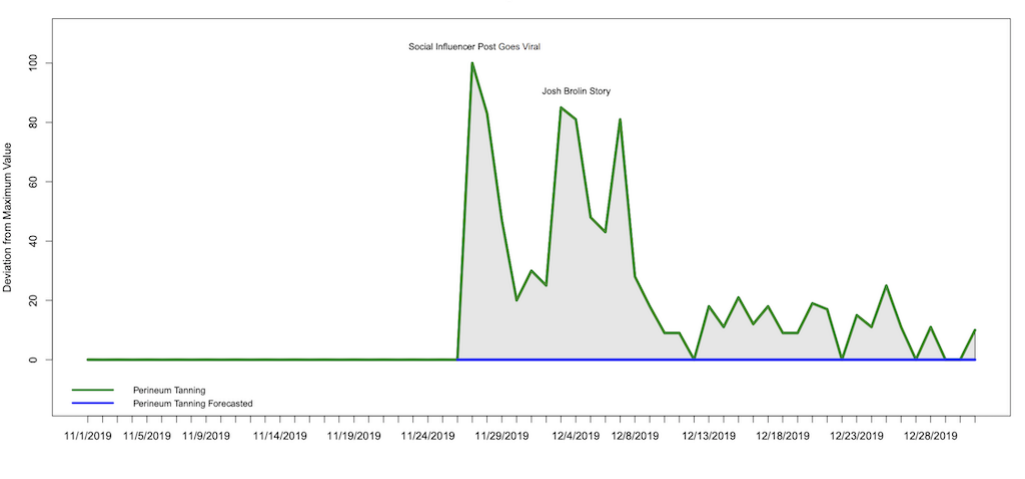
ARIMA (autoregressive integrated moving average)-forecasted trend of “perineum tanning” from November 25 to December 31, 2019 (blue), and actual search trends (green) with the shaded area showing the cumulative area of increase.

The area under the curve (shaded in Figure 2) for “perineum tanning” indicated a cumulative increased interest of 919.00% from the day the post went viral to the end of the year.

**Figure 3:**
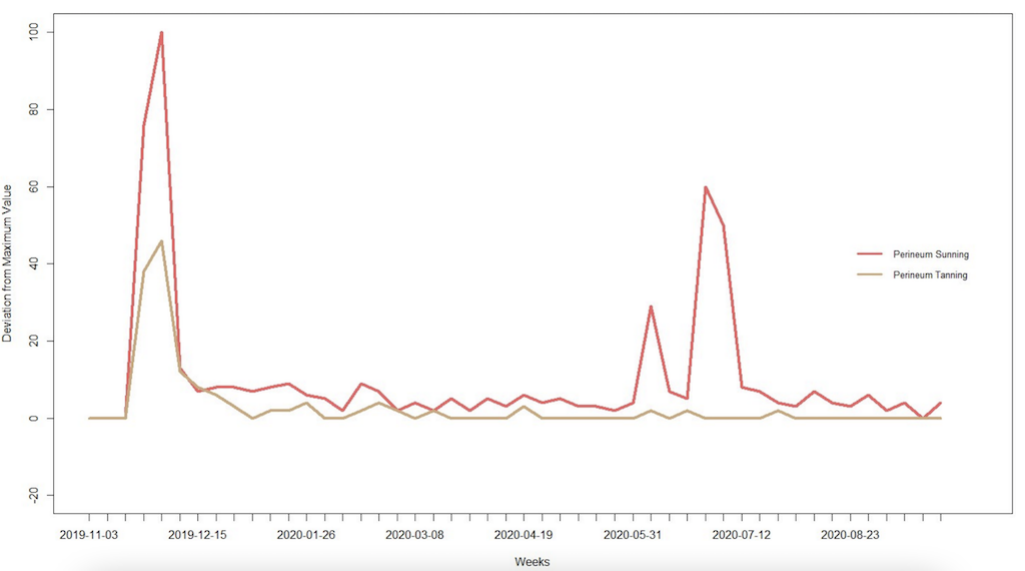
Google Trends analysis of the terms “perineum sunning” and “perineum tanning” from November 1 to September 30, 2020.

Monthly search volumes from UberSuggest show no search interest in “perineum sunning” or “perineum tanning” before the post went viral on November 25, 2019. After the post, search volumes for the search terms “perineum sunning” and “perineum tanning” increased from 0 in October to a combined 52,599 searches for the remaining days in November and climbed to 67,598 searches in December. User engagement showed that nearly half of the individuals who searched for “perineum sunning” or “perineum tanning” were likely to click a returned link for more information. Search trends for perineum sunning showed additional spikes in the week of June 6 (29% of the original search interest peak), June 28 (60% of the original search interest peak), and July 5 (50% of the original search interest peak) (Figure 3).

## Discussion

### Principal Findings

Our results show that one Instagram post, a subsequent viral tweet, and mainstream media coverage generated significant interest in perineum sunning. This new proposed health trend appeared in over 250 articles from numerous media outlets, which spurred others to engage in the behavior. For example, actor Josh Brolin attempted perineum sunning, which resulted in a severe sunburn to his anogenital area. Other iconic figures such as the famous American music producer Diplo, popular health author and entrepreneur Dave Aspery, and American actress Shailene Woodley have all shared their experience of perineum sunning through news media. Despite being deemed a “wellness” technique, the mainstream attention garnered by perineum sunning could lead to harmful health consequences.

Our trends analysis demonstrates continued public search interest in “perineum sunning” and “perineum tanning” for almost a year since the original post on October 21, 2019. Continued search interest in perineum sunning showed a resurgence during the summer months. This finding is concerning because UV exposure increases during the summer, and the solar radiation during these months has the greatest intensities of UV-B [7]. Additionally, Tripathi et al [8] found an increase in the prevalence and costs associated with sunburn-related emergency department visits, especially during the months of June, July, and August. While this study did not explore intent to act, if increased search interest during these summer months resulted in more people attempting this practice, they would be exposing themselves to more dangerous levels of UV radiation. While unsure why other spikes of public interest in perineum sunning occurred, we speculate that continued interest is being generated through social media platforms and ongoing news media coverage. Regardless of the reasons for the increased interest, our study suggests that people are continuing to search for perineum sunning, which may lead to higher rates of cutaneous malignancies and poorer health outcomes if more people attempt this unsafe wellness trend.

Exposure of skin to the UV rays present in sunlight has acute and chronic effects that can occur at doses of UV light that are nonerythemogenic. Short-term effects are sunburn (ranging from solar erythema to vesiculation/bullae formation) and tanning. Long-term effects include photoaging and UV-induced tumor formation [9,10]. It is well known that UV exposure is the most obvious risk factor for cutaneous malignancies such as melanoma, basal cell carcinoma, and squamous cell carcinoma [11]. To make matters worse, melanomas in less visible areas, such as the buttocks and perineum, have worse prognosis independent of tumor characteristics and visibility on self-skin examinations [10]. Furthermore, perineum skin is still vulnerable to risk of sunburn and, over time, cancer formation. To our knowledge, no study has shown perineal skin to have a special ability to generate more vitamin D production than other areas of the skin, nor is there any human evidence that sunning this specific area promotes positive changes in mood, increases libido, and improves regulation of hormones or circadian rhythm. Promotion of health misinformation, such as perineum sunning, via social media is quickly becoming a public health threat.

Non–evidence-based health trends and medical misinformation originating on social media are recognized challenges with serious public health implications (eg, antivaccination campaigns), which can place a significant burden on medical professionals and health care systems in one day, as our results show. Protecting the value of accurate medical information is of utmost importance as science and health information can be strategically manipulated by social media while perpetuating misinformation [12]. Strategies to combat the spread of misinformation require a collaborative approach involving medical journals, researchers, and physicians [13]. Doctors and health care professionals are encouraged to use social media platforms—the source of the majority of misinformation—as educational tools to promote accurate medical information and protect the integrity of online health information [14].

Along with health care professionals, journals must also be proactive in coordinating efforts to address public health misinformation that may harm the general public. We agree with Armstrong et al [15] and recommend journals publish articles with the intention of educating and redirecting public behavior at pertinent times when widespread dissemination of health misinformation has occurred. The *Journal of Medical Internet Research*, and its sister journal *JMIR Dermatology*, are two examples of journals that seem to be dedicated to publishing research focused on combating medical misinformation and raising awareness concerning the quality of health information on the internet [1,16-19]. For example, one study recently published by *JMIR Dermatology* evaluated the quality of sun-protection information by examining the most popular YouTube videos covering sunscreen [16]. Here the authors concluded that content about sunscreen use was often negative or failed to include important sunscreen use recommendations. Low-quality information surrounding sunscreen use, coupled with our study’s results, further demonstrates the need for a collaborative approach to combat medical misinformation, especially in risky sun behaviors.

### Limitations

Our study is limited by its cross-sectional design and should not be generalized. While Google Trends has been used to examine increased public interest and subsequent actions [20,21], our study did not determine intent. Therefore, future research in social media health trends may consider collecting participant surveys of *intent to act* after viewing the post. Lastly, by using Google Trends, we could only calculate relative search volumes.

### Conclusion

Our findings suggest that it took only 24 hours for a potentially dangerous “health” trend to capture the spotlight of mainstream media outlets—an alarming exposé in the power of social media concerning perineum sunning. Additionally, continued observance of the search interest in perineum sunning showed a resurgence during the summer months. Exposure to sunlight is dangerous, and sensitive areas such as the perineum have worse prognosis even when detected during skin examinations. Dermatologists and physicians in other fields of medicine should be aware of perineum sunning and should consider that its popularity may warrant additional inquiry about sun exposure and tanning during patient encounters.

## Abbreviations

ARIMA: autoregressive integrated moving average
